# Psychosocial aspects of quality of life outcomes in post-treatment human papillomavirus-associated cancer survivors in the United States: A scoping review

**DOI:** 10.1177/20551029251327438

**Published:** 2025-03-27

**Authors:** Seiichi Villalona, Aravind Rajagopalan, Qianwei Chen, Julie Sumski, Sharon Manne

**Affiliations:** 1 Department of Medicine, 6572 University of Pennsylvania Perelman School of Medicine, Philadelphia, PA, USA; 2 6572 Hospital of the University of Pennsylvania, Philadelphia, PA, USA; 3 12287 Rutgers Robert Wood Johnson Medical School, Piscataway, NJ, USA; 4 Institute for Health, Health Care Policy and Aging Research, New Brunswick, NJ, USA; 5 Cancer Institute of New Jersey, New Brunswick, NJ, USA

**Keywords:** cancer, stigma, health behaviour, survivorship, clinical health psychology

## Abstract

Human papillomavirus (HPV)-associated cancers (oropharyngeal, cervical, vulvar, vaginal, anorectal, and penile cancers) have previously been reported to have favorable survival outcomes making patients’ quality of life (QoL) an important consideration for clinicians. This scoping review examined the literature on the post-treatment psychosocial QoL outcomes in patients HPV-associated cancers in the United States. The final set of 57 articles were comprised of patients that predominantly identified as Non-Hispanic White, females, or those with cervical or gynecologic cancers. Physical and psychological QoL were the most studied domains. Qualitative studies demonstrated salient themes including low health literacy on HPV-associated cancers, decreased sexual well-being, and increased feelings of stress and fear. Future work is needed in understanding psychosocial QoL in non-gynecologic HPV-associated cancers among individuals from underrepresented racial/ethnic groups, male patients, and those of lower socioeconomic status. Additionally, cancer-related stigma is relatively understudied among patients with HPV-associated cancers.

## Introduction

Human papillomavirus (HPV) is the sexually transmitted infection (STI) with the highest prevalence and incidence in the United States (US), accounting for 13 million new cases per year. Similarly, high rates are seen worldwide, with a global pooled prevalence of 31% for any genital HPV and 21% for high-risk HPV(Bruni et al., 2023). HPV infection, primarily with the high-risk types 16 and 18, has been associated with oropharyngeal, cervical, vulvar, vaginal, anorectal, and penile cancers (Hirth, 2019). In the US, cervical and oropharyngeal cancers (OPCs) remain the most common HPV-associated malignancies in women and men, respectively (Liao et al., 2022). The main risk factors for developing cervical cancer among women include having multiple sexual partners, having oral sex, and being immunosuppressed (Moscicki and Palefsky, 2011; Stanley et al., 2012). In contrast, OPCs, particularly HPV-associated OPCs (HPV-OPCs), are distinct entities that have been associated with the number of oral sex partners (Drake et al., 2021). However, the relationship between oral HPV infection and oropharyngeal squamous cell carcinoma (OPSCC) is complex, with nuances related to the timing, intensity of oral sex, and the type of sexual partner, further impacting the risk (Drake et al., 2021). The natural history of OPSCC differs from that of cervical cancer, with certain pre-lesions seen in cervical cancer that are absent in OPC. This makes the trajectory of OPSCC development less clear (Agalliu et al., 2016). While certain studies have indicated a higher prevalence of oral HPV infections among men who have sex with men, it is important to note that a direct association between sexual orientation and increased risk of HPV-OPC has not been conclusively established (Drake et al., 2021; Heck et al., 2010).

The epidemiological history of HPV-associated cancers and vaccination is important in contextualizing how the burden of these malignancies has shifted in affecting different demographic groups in the US. OPSCC was first recognized in 2009. In 2006, the Food and Drug Administration (FDA) recommended vaccination against high-risk HPV infection to decrease cervical cancer rates. The FDA’s Advisory Committee on Immunization Practices (ACIP) initially approved Merck Gardasil®, as a three-dose schedule for females aged 9-26 in 2006 for protection against four aggressive HPV strains (Slade et al., 2009). Gardasil® was later approved for males aged 13-21, as well as up to age 26 in high-risk male individuals; vaccination for all males up to age 26 was later approved in 2015 (Centers for Disease Control and Prevention, 2021a; Daley et al., 2017). In 2015, the FDA approved Merck’s multivalent version of the Gardasil® vaccine, which covered five additional oncogenic HPV strains (Markowitz et al., 2018; Slade et al., 2009). By 2018, vaccination was approved for both women and men ages 27-45 (Markowitz et al., 2018; Slade et al., 2009).

Public health campaigns to prevent cervical cancer through vaccination and enhanced screening have significantly reduced its age-adjusted incidence and disease burden (Van Dyne et al., 2018). In contrast, HPV-OPCs have seen a marked rise, especially in males. The incidence of OPC in men has recently exceeded that of cervical cancer in women, though the exact timing of this shift differs among studies (Centers for Disease Control and Prevention, 2021a; Centers for Disease Control and Prevention, 2021b; Lechner et al., 2022; Van Dyne et al., 2018). While cervical cancer and OPCs are the primary HPV-associated malignancies in women and men, respectively (Liao et al., 2022), HPV-OPCs in males now represents the most widespread HPV-linked cancer (Centers for Disease Control and Prevention, 2021a). HPV-OPCs currently affect younger individuals with fewer behavioral risk factors, such as heavy tobacco or alcohol consumption. HPV-associated anorectal cancers predominantly impact female patients (Liao et al., 2022; Lin et al., 2019; Saleem, 2011), and individuals with compromised immune systems, especially those with human immunodeficiency virus (HIV) (Walsh et al., 2015; Ye et al., 2020). Moreover, young individuals of color identifying as sexual and gender minorities face a higher incidence and prevalence of HIV (Koenig et al., 2016), which is a known risk factor for reduced HPV viral clearance and subsequent anorectal cancer development (Walsh et al., 2015; Ye et al., 2020).

Previous research indicated that HPV-associated cancers yield better health outcomes than non-virally mediated malignancies in similar anatomical sites (Guerendiain et al., 2022; Kugelman et al., 2022; Wang et al., 2015). Given their favorable prognosis and younger patient demographics, post-treatment survivorship and quality of life (QoL) become crucial. QoL, encompasses daily physical, emotional, and social functions reflects the interplay between a disease’s physiological factors and patients’ psychological experiences (Penson et al., 2006; Wilson and Cleary, 1995). These cancers, tied to behavioral risk factors like sexual activity, offer unique insights into patient QoL. Few studies have explored psychosocial distress in HPV-associated cancer patients. Compared to the general populace, OPC patients face higher depression and anxiety risks (Qualliotine et al., 2017), and exhibit reduced QoL (Hammermüller et al., 2021). Notably, previous research suggests HPV-positive oropharyngeal cancer patients experience more depressive symptoms than their HPV-negative counterparts (Bauman et al., 2016; Dodd et al., 2019). Studies also highlight the shame, embarrassment and guilt experienced among patients with HPV-associated anogenital and gynecological cancers (Leppard, 2016; Longabaugh, 2017; McCaffery et al., 2006).

This scoping review aimed to examine the existent literature and evaluate the current body of research on the QoL outcomes studied in patients with HPV-associated cancers, particularly focusing on the psychosocial needs of this unique cancer patient population. Through this scoping review, we aimed to identify areas for further research regarding psychosocial QoL outcomes among this patient population during and after oncological treatment.

## Methods

Scoping review methodology offers researchers a key approach in examining both the breadth and depth of the scientific topic of interest (Arksey and O’Malley, 2005). They provide a comprehensive overview through identifying gaps within the literature and topical areas of scholarly engagement without being exhaustive or too cursory (Arksey and O’Malley, 2005; Peters et al., 2015). The frameworks of Arksey and O’Malley (2005) and Levac et al. (2010) were used to guide this scoping review.

### Identifying research questions

This scoping review aimed to assess psychosocial QoL outcomes that have been studied, the methodologies employed, and their contexts. Due to the established link between HPV and the majority of anorectal, gynecologic, oropharyngeal, and penile cancers and the introduction of its vaccine, our emphasis was on studies from the post-vaccine era (post-2006).

The guiding research questions explored in this review included: (1) which psychosocial QoL outcomes have been studied and what measures, assessments, or tools have been used to examine these outcomes?; (2) what proportion of these studies included participants of both sexes as well as populations of color?; (3) what analytic approaches have been employed (qualitative, quantitative, mixed-methods)?; and (4) from a geographic perspective, where have these studies been conducted in the US? This broad search approach strategy was used in conceptualizing the present scoping review, since to our knowledge, no prior published work has specifically examined these topics among survivors of HPV-associated cancers.

### Identifying relevant studies

Four databases were searched in this scoping review which included: PubMed, Scopus, Web of Science, and CINAHL (Cumulative Index to Nursing and Allied Health Literature). The Boolean search term combinations were created using the following template: “HPV” and “*[outcome of interest]*” and “*[cancer type]*”. Examples of search term combinations include: “‘HPV’ and ‘quality of life’ and ‘anal cancer’”; “‘HPV’ and ‘stigma’ and ‘cervical cancer’”; “‘HPV’ and ‘depression’ and ‘oropharyngeal cancer’”; “‘HPV’ and ‘anxiety’ and ‘penile cancer’”; and “‘HPV’ and ‘sexual function’ and ‘vulvar cancer’”. A full list of search terms and their combinations are listed in Supplemental 1.

### Study selection

We limited our review to peer-reviewed studies that met the following criteria: (1) based in the US; (2) written in English; (3) analyzed primary or secondary data; (4) were published January 01, 2007 to November 30, 2023; and (5) included adults (>18 years of age) diagnosed with an HPV-associated cancer. Studies were excluded if they were: (1) abstracts presented at scientific meetings, (2) dissertations/theses; (3) basic science studies involving non-human subjects research conducted in laboratory settings; (4) editorials or opinion pieces; or (5) other literature reviews of data syntheses.

**Figure 1.**
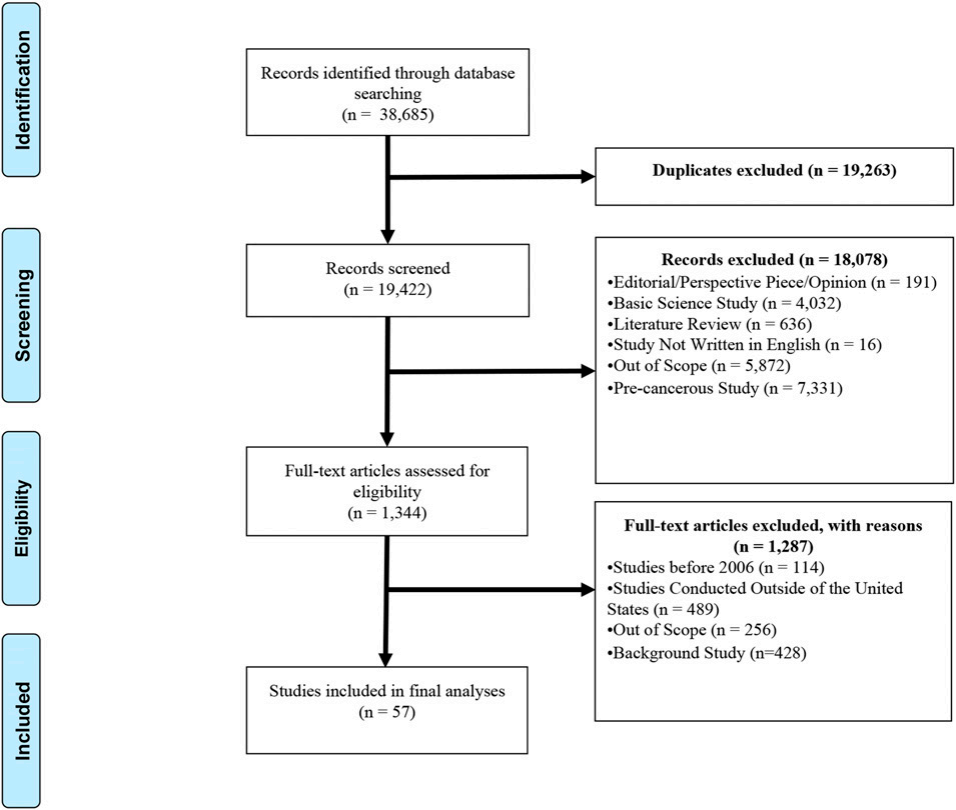
PRISMA flow diagram of article selection process.

The final list of combined Boolean search terms generated an initial set of articles imported into an EndNote library (n = 38,685) for screening. After the removal of the duplicates (n = 19,263), the study team (SV, QC, JS, and AR) initially screened the abstracts from the remaining articles (n = 19,422) and coded each article using the inclusion and exclusion criteria (Figure 1). In this phase, each article was screened independently by two team members. A third team member reconciled coding differences. This methodological approach minimized individual biases from each team member in the screening process. The remaining articles (n = 1344) were subjected to full-text reviews using the same approach as the screening phase, whereby a final set was generated for the analyses (n = 57). Figure 1 outlines the study selection methodology in the form of a PRISMA flow diagram.

### Data extraction

General information extracted from each article included the author, publication year, study type, geographic location of study, demographics (if available) of the study population, study outcomes, and main study findings. Each article was read by one member of the research team and reviewed by another member during team meetings where discussions focused on thoroughly assessing the trends and broader themes of the literature.

## Results

### Demographics by sex, race, and geographic region

**Figure 2.**
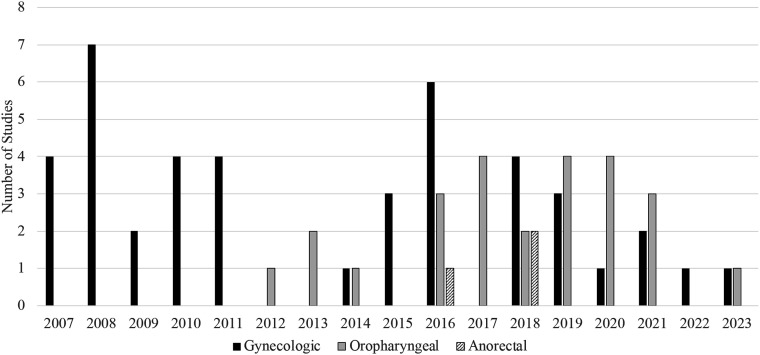
Types of HPV-associated cancer studies by year.

A final set of 57 articles were included in the analyses, comprising of 333,665 participants. Table 1 outlines the major findings of the studies included in this scoping review. Most of the articles focused on cervical or gynecologic cancers (53%, n = 30) followed by OPC (40%, n = 23). Studies that included patients with anorectal cancer represented 5% (n = 3) of the final set of articles. No studies in the final set included or focused on patients with penile cancer. Of the 57 studies, 54 (95%) included patients with only one type of HPV-associated cancer, whereas the remaining three studies (5%) looked at more than one type (e.g., cervical, anorectal, and oropharyngeal cancer). Most studies were localized to a geographic region: 86% (n = 49) were centered in a single metropolitan area or were conducted in a single state, and 5% (n = 3) looked at regions within the US such as the Northeast, Midwest, or Southwest. Nine percent (n = 5) were nation-wide studies. During the time period included in this scoping review, there was a larger emphasis on gynecological HPV-associated cancers between 2007-2016, which declined in the subsequent years (Figure 2). Before 2012, there were no published studies on HPV-associated OPCs, which rose and comprised most studies after 2012 (Figure 2). Studies on HPV-associated anorectal cancers similarly did not appear until 2016.

**Table 1.** Summary of findings from studies examining quality of life (QoL) in patients diagnosed with HPV-associated cancers.

Title & Year	Cancer type	N	Methods	Sexes studied	Global QoL	Physical QoL	Sexual function QoL	Psychological QoL	Social QoL	Financial QoL
(Donovan et al., 2007)	Cervical	100	Quantitative	Females	+	+	+			+
(Greenwald and McCorkle, 2007)	Cervical	208	Quantitative	Females	+				+	+
(Lindau et al., 2007)	Gynecologic	1239	Mixed	Females	+		+			+
(Ashing-Giwa, 2008)	Cervical	23	Mixed	Females	+	+	+	+		+
(Baze et al., 2008)	Cervical	1	Qualitative	Females	+	+	+	+	+	
(Carter et al., 2008)	Cervical	30	Quantitative	Females	+	+	+	+		
(Clemmens, 2008)	Cervical	19	Qualitative	Females	+					
(Gotay et al., 2008)	Cervical	41	Quantitative	Females	+	+	+	+	+	
(Greenwald and McCorkle, 2008)	Cervical	179	Quantitative	Females	+	+	+	+		+
(Greenwald et al., 2008)	Cervical	740	Quantitative	Females	+	+				+
(Ashing-Giwa et al., 2009)	Cervical	560	Quantitative	Females	+	+	+	+	+	+
(Bartoces et al., 2009)	Cervical	145	Quantitative	Females	+	+		+	+	+
(Carter et al., 2010)	Cervical	71	Quantitative	Females	+	+	+	+		
(Dyer, 2010)	Cervical	19	Qualitative	Females	+				+	
(Hill et al., 2011)	Gynecologic	261	Quantitative	Females			+			
(Linsky et al., 2011)	Multiple	1670	Quantitative	Both	+	+				
(Moreno et al., 2012)	Oropharyngeal	44	Quantitative	Both			+			
(Baxi et al., 2013)	Oropharyngeal	10	Qualitative	Males			+	+		
(Milbury et al., 2013)	Oropharyngeal	62	Mixed	Both						
(Maxwell et al., 2014)	Oropharyngeal	177	Quantitative	Both	+	+		+		
(Osann et al., 2014)	Cervical	204	Quantitative	Females	+	+	+	+	+	
(Chan et al., 2015)	Cervical	228	Quantitative	Females			+			
(Kim et al., 2015)	Cervical	88	Quantitative	Females	+	+				
(Wenzel et al., 2015)	Cervical	204	Quantitative	Females	+	+	+	+		
(Baxi et al., 2016)	Oropharyngeal	102	Quantitative	Both	+	+	+	+		
(D’souza et al., 2016)	Oropharyngeal	48	Quantitative	Both		+				
(Doll et al., 2016)	Gynecologic	185	Mixed	Females	+	+		+		
(DuHamel et al., 2016)	Anorectal	70	Quantitative	Females	+		+	+		+
(Fleming et al., 2016)	Cervical	32	Quantitative	Females	+	+	+		+	
(Iyer et al., 2016)	Cervical	204	Quantitative	Females	+			+	+	
(Ling et al., 2016)	Oropharyngeal	92	Quantitative	Both	+	+		+		
(Westin et al., 2016)	Gynecologic	1029	Quantitative	Females		+	+	+		
(Goepfert et al., 2017)	Oropharyngeal	935	Quantitative	Both	+	+		+	+	
(Qualliotine et al., 2017)	Oropharyngeal	65	Quantitative	Both	+			+		
(Taberna et al., 2017)	Oropharyngeal	262	Quantitative	Both			+		+	
(Xiao et al., 2017)	Oropharyngeal	720	Quantitative	Both	+	+				
(Baxi et al., 2018)	Oropharyngeal	185	Quantitative	Both	+	+	+	+		
(Wilford et al., 2018)	Cervical	204	Quantitative	Females				+		
(Wo et al., 2018)	Multiple	107	Quantitative	Both			+			
(Best et al., 2018)	Multiple	27	Qualitative	Both	+			+	+	
(Hoover et al., 2019)	Cervical	12	Qualitative	Females	+				+	
(Hubbs et al., 2019)	Gynecologic	85	Quantitative	Females	+		+			
(Janz et al., 2019)	Oropharyngeal	38	Quantitative	Both				+		
(Massa et al., 2019)	Oropharyngeal	261,631	Quantitative	Both	+	+				+
(Windon et al., 2019)	Oropharyngeal	150	Quantitative	Both	+	+				
(Mady et al., 2019)	Oropharyngeal	104	Quantitative	Both	+	+		+		+
(Beeler et al., 2020)	Oropharyngeal	63	Mixed	Both				+		+
(Kompelli et al., 2020)	Oropharyngeal	39	Quantitative	Both		+			+	+
(Rhoten et al., 2020)	Oropharyngeal	81	Mixed	Both	+	+	+			
(Wilson et al., 2020)	Gynecologic	20	Mixed	Females		+	+			
(Xu et al., 2020)	Oropharyngeal	76	Quantitative	Both	+	+	+	+		+
(Bai et al., 2021)	Cervical	84	Quantitative	Females	+	+	+	+	+	
(Cardoso et al., 2021)	Oropharyngeal	892	Quantitative	Both	+	+		+		
(Kaffenberger et al., 2021)	Oropharyngeal	73	Quantitative	Both		+		+		
(Coleman et al., 2022)	Cervical	91	Mixed	Females	+	+	+	+	+	
(Avila et al., 2023)	Gynecologic	15	Mixed	Females	+	+		+	+	
(Chakoma et al., 2023)	Oropharyngeal	60,361	Quantitative	Both				+

Approximately half of the studies (54%; n = 31) exclusively recruited female patients. Twenty-three (40%) of the studies were conducted among patients of both sexes. Only one study (2%) exclusively recruited male patients. Within the subset of studies that examined patients with OPCs (n = 23), 65% of participants were male (n = 213,245), and 36% were female (n = 105,394). The only study conducted among patients with anorectal cancer exclusively recruited female patients. One qualitative study on emotions and fears associated with a diagnosis of OPC consisted only of male participants, whereas another study on anorectal cancer assessed only female individuals’ sexual QoL.

**Figure 3.**
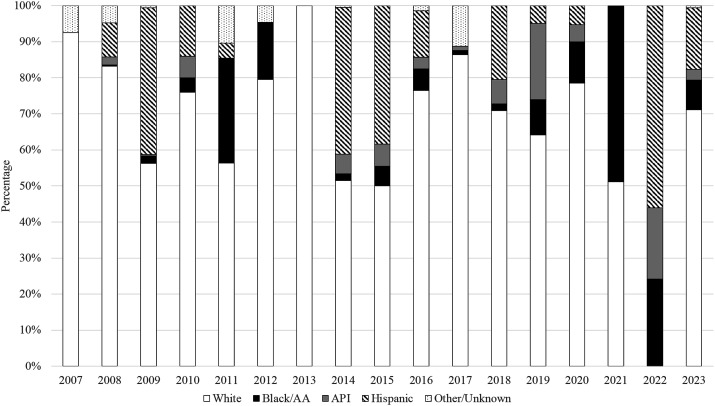
HPV-associated cancer studies by race/ethnicity.

Of all studies that reported a breakdown of participants by ethnic or racial group, an aggregate of 64% of participants identified as Non-Hispanic White (n = 214,989). The aggregate sample was represented by 17% (n = 57,167) of participants that identified as Asian American/Pacific Islanders, 9% (n = 30,897) Black/African American, and 7% (n = 24,073) Hispanic/Latino. Among the studies conducted in patients with gynecologic cancers, 63% identified as Non-Hispanic White (n = 171,751), 20% as Asian American/Pacific Islander (n = 55,384), 9% as Black/African American (n = 25,975), and 5% as Hispanic/Latino/a (n = 13,745). For studies among those with OPC that provided a breakdown of race or ethnicity, aggregate proportions were represented by 65% Non-Hispanic White (n = 214,041), 17% Asian American/Pacific Islander (n = 57,162), 9% Black/African American (n = 30,834), and 7% Hispanic/Latino/a (n = 23,735). Figure 3 outlines the breakdown of participants in the included studies by race/ethnicity. While those that identify as Non-Hispanic White represent most of the sample, fluctuations in proportions were seen among other racial and ethnic groups across studies (Figure 3).

### Analytical methods and outcome measures

Among the 57 final articles, 73% (n = 42) relied on quantitative methodologies such as survey research and employed regression modeling to assess patient QoL. Mixed quantitative and qualitative methods were employed by 16% (n = 9) and 11% (n = 6) of studies, respectively. Surveys or questionnaires used by both subsets were validated tools used in prior studies assessing different aspects of QoL, including global QoL, physical QoL, psychological QoL (depression, anxiety), sexual intimacy and function, social QoL, and financial QoL.

Studies assessing the domain of global QoL employed the following instruments: Functional Assessment of Cancer Therapy (FACT-G or its cancer-specific variations, n = 11), European Organization for Research and Treatment of Cancer Quality of Life Questionnaire (EORTC-QLQ, n = 6), Short Form Survey developed by the Medical Outcomes Study (SF-12 or 36, n = 4), and University of Washington Quality of Life Questionnaire (UW-QoL, n = 4). Physical QoL was assessed with the following instruments: FACT-G (n = 11), SF-12 (n = 4), UW-QoL (n = 4), the EuroQol 5-dimension Questionnaire (EQ-5D, n = 3), and the Quality of Life Patient/Cancer Survivor Version Scale (QOL-CSV, n = 1). Psychological QoL was examined with the following instruments: FACT-G (n = 8), SF-12 or 36 (n = 4), UW-QoL (n = 4), Brief System Inventory 18 (BSI-18, n = 5), Center for Epidemiologic Studies Depression Scale (CES-D, n = 4), and the Patient Health Questionnaire-9 (PHQ-9, n = 2). Sexual QoL was surveyed with the Female Sexual Function Index (FSFI, n = 7), the Sexual Adjustment Questionnaire (SAQ, n = 6), and the Gynecologic Problem Checklist (GPC, n = 3). Social QoL was examined in studies that used the Medical Outcomes Social Support Survey (MOSS, n = 3), and items from the SF-12 or 36 (n = 3). Studies assessing financial QoL utilized elements from the EORTC-QLQ (n = 4), MOSS (n = 3), and the Comprehensive Score for Financial Toxicity (COST, n = 2). Among the studies included in this scoping review that employed quantitative methods, most used regression modeling to predict outcomes on QoL measures based on pre-existing conditions at the time of assessment. Quantitative studies typically sought to establish prevalence of various QoL outcomes among long-term cancer patients and survivors.

Qualitative studies (n = 6) represented 11% of the final set of included articles. These studies employed methodologies such as participant interviewing and thematic analyses. Through semi-structured or unstructured interviews, study authors elicited patient narratives to form the basis for needs assessments and hypothesis generation. Across qualitative studies or mixed methods studies that utilized more than one interviewee, thematic analysis identified common themes or phenomena across multiple participants’ responses regarding their QoL outcomes. Mixed methods studies generally relied more on survey data similar to quantitative studies and supplemented with anecdotal evidence from patient interviews to generate conclusions. Across all quantitative, qualitative, and mixed methods studies, 18 studies specifically incorporated author-designed surveys or interview questions.

Global QoL was assessed in a majority of studies (72%, n = 41). Among the subtypes of QoL, physical QoL was the most common outcome (63%, n = 36), of which pain and fatigue were the most surveyed physical symptoms. Sexual QoL outcomes was the next most commonly studied outcome, which primarily focused on sexual function (49%, n = 28). Psychological QoL was assessed in 58% of studies (n = 33) as either anxiety (49%, n = 28) or depression (46%, n = 26). Financial QoL (25%, n = 14), such as socioeconomic status (SES), insurance status, and financial toxicity, and social QoL (30%, n = 17) such as social support were the least common outcomes assessed. Three out of six studies assessing stigma were qualitative studies that drew from patient perspectives and interviews.

Qualitative and mixed methods studies highlighted several key findings on psychosocial outcomes from patient interviews. Firstly, there was a notable knowledge gap concerning HPV-associated cancers. Patients did not know that their cancers were caused by or strongly associated with prior HPV infection. Secondly, the significance of physician-patient communication was underscored. Two studies showed that improved communication could enhance prevention rates and patient comprehension of long-term management. Thirdly, studies emphasized the substantial impact on sexual function and intimacy among HPV cancer survivors. The lack of early discussions on these issues with physicians was identified as an area in clinical medicine needing improvement to significantly lower sexual health-related morbidity. Another study emphasized reinterpreting the survivorship experience to positively influence patient QoL. Fourthly, considerable stigma, stress, and fear were linked to HPV-associated cancers, often rooted in the guilt, shame, and embarrassment about acquiring the infection through sexual behavior. Lastly, financial burdens associated with HPV cancer treatments emerged as a concern, potentially affecting long-term QoL and treatment adherence.

## Discussion

### Research gaps among demographic groups

This scoping review highlights the current foci of HPV-associated cancer survivor subgroups included within the QoL literature and the QoL domains examined. Our findings show that the current body of literature has mainly focused on individuals diagnosed with HPV-associated cervical or gynecologic cancers (53%, n = 30), and predominantly recruited patients identifying as non-Hispanic White (64%) or exclusively female (54%, n = 31). Racial/ethnic minorities, males, and other types of HPV-associated cancers are largely underrepresented in research, and SES is not well-characterized.

### Underrepresented patient populations by race/ethnicity & sexual orientation

The underrepresentation of racial/ethnic minorities and underserved populations in cancer research has previously been identified as a pressing challenge for the field of oncology (Simon et al., 2014). Biomedical oncologic research has shown that when looking at over 230,000 biospecimens collected from cancer patients, 88% of the samples originated from patients who self-identified as non-Hispanic White. Our findings mirror this disparity and highlight the underrepresentation of ethnic/racial minorities in socio-behavioral research focusing on QoL of patients with HPV-associated cancers. Although the final set of articles reviewed had wide racial diversity with most of the studies including some patients of color, these groups still generally represented small proportions of participants across these studies. The underrepresentation of racial/ethnic groups within the QoL literature regarding HPV-associated cancers is further magnified when considering that the average annual percentage change (AAPC) in incidence was increasing significantly among particular patient subgroups and cancer types (Liao et al., 2022). For example, while anorectal HPV-associated cancers increased in both non-Hispanic White and Black patients from both sexes, non-Hispanic Black males represented the demographic subgroup with the largest increase in AAPC (Liao et al., 2022). The findings from our scoping review and the continuing demographic changes in the epidemiology of HPV-associated cancers in the US collectively call for future research exploring the QoL of patients with these cancers, particularly those of underrepresented racial/ethnic groups.

Another underrepresented demographic group worth noting was individuals with HPV-associated cancers that identify as sexual minorities. Only one qualitative study specifically analyzed the effect of sexual orientation on patients’ experiences with these cancers (Best et al., 2018). Research has shown that individuals who identify as lesbian, gay, or bisexual (LGB) have significantly higher odds of being diagnosed with cancer, regardless of type, when compared to their heterosexual counterparts of the same sex at birth (Gonzales and Zinone, 2018). Patients who identify as sexual minorities have been found to have increased risks of developing HPV-associated cancers (Quinn et al., 2015). Bisexual females face higher rates of cervical cancer than their heterosexual counterparts (Boehmer et al., 2011). Transgender men and women have been identified as a neglected population with respect to surveillance, prevention, and screening of HPV-associated cancers, making them one of the demographic subgroups at higher risk (Brown et al., 2017). Taken together, the notable underrepresentation of sexual minorities in HPV-associated cancer research warrants future work to include this uniquely vulnerable population to be included in QoL studies.

### Patient populations of lower SES and risk of financial toxicity

Regarding financial QoL, only 25% (n = 14) of the studies included in this review reported or examined participant SES, detailing participants’ insurance and/or income status. Despite the advent of modern oncology therapies improving overall health outcomes, these advancements carry significant financial implications for patients, paralleling the physical and psychological challenges post-diagnosis. Approximately 22% to 64% of cancer patients express concern about paying their medical bills (Ell et al., 2008) and they are 2.5 times more likely to face bankruptcy than their healthy counterparts (Ramsey et al., 2013). Income has been previously identified as a predictor of overall QoL, influencing patients’ physical, emotional, and role functioning (Roick et al., 2019). Recent oncology literature has discussed *financial toxicity,* which is defined as the monetary burden and downstream consequences of receiving healthcare for complex medical conditions like cancer. These studies revealed an association between cancer patients reporting financial toxicity and decreased health-related QoL, reduced survival rates, and diminished treatment adherence (Lathan et al., 2016; Neugut et al., 2011; Ramsey et al., 2016; Zafar et al., 2015). Given that individuals with lower SES tend to have a limited awareness of HPV and its link to cancer (Chido-Amajuoyi et al., 2020), our findings suggest that financial QoL and financial toxicity in HPV-associated cancer patients is an under-researched yet critical area for future studies.

### Sex at birth

The majority of studies (54%, n = 31) in the final set recruited and included exclusively female patients. Only one study exclusively included male patients with OPC (Baxi et al., 2013). Among the studies that included both males and females, males comprised 65% (n = 213,761) of the study population. When looking at the epidemiologic trends of HPV-associated cancers between 2001 and 2017 across all anatomic sites (including malignancies affecting the reproductive organs of both sexes), female patients represented approximately 60% of all virally related cases of which 71% were gynecologic cancers (cervical, vaginal, or vulvar) (Liao et al., 2022). It is important to note that while screening methods for HPV-associated gynecologic cancers, particularly cervical cancer, exist and are relatively efficient due to the identifiable precancerous lesions, there is currently no established screening method for OPC. This is largely because OPC lacks recognizable precancerous lesions, making developing efficient screening strategies challenging (Kepka et al., 2016). A recent study by Liao et al. (Liao et al., 2022) reported a significant increase in the AAPC in all HPV-associated cancers among male patients, particularly oropharyngeal and anorectal cancers. As previously discussed, the landscape of HPV-associated cancers is changing such that male patients represent an increasing proportion of those affected by these virally related malignancies (Centers for Disease Control and Prevention, 2021a; Centers for Disease Control and Prevention, 2021b; Liao et al., 2022; Van Dyne et al., 2018). The observed epidemiological shift in OPC may be influenced by several factors: the rise in HPV infection rates, including potentially long-standing oral infections; the success of cervical cancer screening and vaccination initiatives affecting cervical cancer trends; and traditional risk factors like alcohol and smoking. While cervical cancers have shown changes possibly linked to interventions, HPV-associated OPC has a longer development timeline, making the immediate impact of preventative measures less evident. Additionally, it is worth noting that screening for precancerous oropharyngeal lesions is currently not feasible (Chaturvedi et al., 2018; Finneran et al., 2021; Osazuwa-Peters et al., 2017). Collectively, the findings from this scoping review additionally highlight the relatively understudied area of QoL research, particularly among males with HPV-associated cancers.

### Heterogeneity of QoL measures

This scoping review evaluated the aspects of QoL and the measures used to study patients with HPV-associated cancers. The primary domains of QoL investigated were: global, physical, sexual function, psychological, social, and financial. Notably, most studies assessed the physical (63%, n = 36) or sexual function (49%, n = 28) domains of QoL. Of the 28 studies focusing on sexual function, 20 (71%) exclusively included female patients with HPV-associated cancers, predominantly those with gynecologic cancers. Among the seven studies that examined sexual function in both male and female patients, six were among those with OPCs. Notably, no studies in our final set of articles addressed the psychosocial QoL of males with penile cancers. This aligns with the relative rarity of penile cancers among all of the HPV-associated cancers (Liao et al., 2022). Our findings underscore the research gap in understanding sexual function in males with HPV-associated cancers across all anatomical sites and in patients of both genders with virally mediated anorectal cancers.

Furthermore, psychological QoL was predominantly gauged using surrogate markers for anxiety, depression, and stress rather than established measures. These markers are inherently symptomatic, so their precision in identifying genuine clinically significant psychological distress or mood disorders may be limited. Our findings align with recent work by Silver et al. (Silver et al., 2023), which underscored a paucity of research specifically focusing on the psychosocial aspects of quality of life in OPC patients. They highlighted the importance of addressing these patients’ emotional, social, and mental well-being, reporting that many past studies were geographically limited and varied in their methodological approaches. This further emphasizes the need for consistent and comprehensive psychosocial QoL measures in the context of HPV-associated cancers.

Among the studies included in this scoping review, the measures employed in assessing psychological QoL ranged from elements borrowed from general QoL questionnaires such as the FACT-G, SF-12/36, and UW-QoL, or specific psychological QoL surveys such as the BSI-18, CES-D, and PHQ-9. The heterogeneity and varying degrees of depth between instruments made it difficult to assess specific psychological QoL across multiple studies. Aspects of psychological QoL that warrant further exploration are the rates of psychological distress and clinically significant mood-related psychiatric disorders among patients diagnosed with HPV-associated cancers using validated psychiatric diagnostic tools, such as the PHQ-9 and GAD-7.

### Exploring the role of stigma & social support

Of the articles included in this scoping review, only five addressed stigma’s impact on patients’ QoL. In the broader cancer patient population, stigma prevalence varies from 18 to 80% (Cho et al., 2013; Ernst et al., 2017; Phelan et al., 2013; Yilmaz et al., 2020). Over 30% of cancer survivors harbor negative views towards their cancers, and about 10% feel discriminated against due to their diagnosis (Cho et al., 2013). The challenge in studying this area lies in its complexity and the diverse methods of measuring cancer-related stigma across studies (Fujisawa and Hagiwara, 2015; Huang et al., 2021). Certain cancer patient subgroups, such as male patients, individuals with lower SES, and those with concurrent mood-related disorders (i.e., depression or anxiety), tend to experience heightened stigma (Huang et al., 2021). This review highlights the existing gap in the literature addressing stigma in HPV-associated cancer patients.

Both tobacco-associated and HPV-associated cancers are tied to individual behavioral risk factors. The stigma surrounding lung cancer, often associated with smoking, has been well documented (Brown Johnson et al., 2014; Chambers et al., 2015; Ostroff et al., 2019). Confirmed HPV infection leads to higher stigma levels, largely attributed to HPV being an STI and the subsequent perception of promiscuity (Daley et al., 2015; Shepherd and Gerend, 2014). Furthermore, increased public awareness due to HPV vaccine promotions has intensified stigmatization among HPV-associated cancer patients (Bachman et al., 2018; Wearn and Shepherd, 2020). Past research indicates these patients are often perceived as unwise, dishonest, and unclean compared to patients with other cancers (Shepherd and Gerend, 2014). Much like lung cancer patients, those with HPV-associated cancers are often perceived as responsible for their condition due to assumed risky behaviors (Lebel and Devins, 2008; Lebel et al., 2013). Given these factors, future research on HPV-associated cancer patients should prioritize understanding stigma, especially considering their predisposition to psychosocial distress.

Lastly, 30% (n = 17) of the studies addressed social support as a QoL aspect post-diagnosis. Given the associated stigma with HPV, there is a pressing need for further research into how these patients perceive and seek social support following diagnosis.

### Study limitations

There are several limitations present in this study. As the goal of this scoping review was to provide a general overview of the literature on HPV-associated cancers and identify topic areas for future research questions, the final set of articles analyzed was produced with liberal exclusion criteria and was not as exhaustive as those found in systematic reviews. Individual rater biases were attempted to be minimized with the screening and iterative methodology. The present review excluded studies conducted at institutions or populations outside of the US. These studies were excluded to minimize the heterogeneity in the patient populations when conducting our analyses.

## Conclusions

To the best of our knowledge, this is the first scoping review examining which psychosocial aspects of QoL have been studied in patients with HPV-associated cancers. The current body of research in this area is primarily focused on non-Hispanic White female patients diagnosed with cervical or other gynecologic cancers. Our analyses identified significant gaps in this literature, particularly regarding the lack of inclusion of patients from underrepresented racial/ethnic groups, males, and individuals of lower socioeconomic status. The studies included in this review demonstrate a broad range of QoL measures with differing domains of focus. This heterogeneity presents a challenge when drawing meaningful comparisons across multiple studies. It underscores the need for more standardized methodologies and measures to facilitate an integrated and comprehensive understanding of QoL among patients with HPV-associated cancers. Finally, despite HPV being the most common STI in the US and the associated stigmatization from the virus, the examination of cancer-related stigma is notably sparse in the context of HPV-associated cancers. Future research should also include a more nuanced exploration of the psychological, social, and financial aspects of QoL. Comprehensive and inclusive studies will enable the development of more targeted and effective interventions to improve the QoL of all patients with HPV-associated cancers.

## Supplemental material

Supplemental Material - Psychosocial aspects of quality of life outcomes in post-treatment human papillomavirus-associated cancer survivors in the United States: A scoping review Supplemental Material for Psychosocial aspects of quality of life outcomes in post-treatment human papillomavirus-associated cancer survivors in the United States: A scoping review by Seiichi Villalona, Aravind Rajagopalan, Qianwei Chen, Julie Sumski and Sharon Manne in Health Psychology Open

## ORCID iD

Seiichi Villalona https://orcid.org/0000-0003-2442-576X

## Statements and Declarations

## References

[bibr1-20551029251327438] Agalliu I Gapstur S Chen Z , et al. (2016) Associations of oral α-β-and γ-human papillomavirus types with risk of incident head and neck cancer. JAMA Oncology 2: 599–606. 26794505 10.1001/jamaoncol.2015.5504 PMC4956584

[bibr2-20551029251327438] Arksey H O’Malley L (2005) Scoping studies: towards a methodological framework. International Journal of Social Research Methodology 8: 19–32.

[bibr3-20551029251327438] Ashing-Giwa KT (2008) Enhancing physical well-being and overall quality of life among underserved Latina-American cervical cancer survivors: feasibility study. Journal of Cancer Survivorship 2: 215–223. 18712607 10.1007/s11764-008-0061-2

[bibr4-20551029251327438] Ashing-Giwa KT Tejero JS Kim J , et al. (2009) Cervical cancer survivorship in a population based sample. Gynecologic Oncology 112: 358–364. 19059636 10.1016/j.ygyno.2008.11.002

[bibr5-20551029251327438] Avila A Cordero J Ibilah O , et al. (2023) Hispanic survivors and caregivers of human papillomavirus–associated cancers: lived experiences in a US–Mexico border community. Health Education & Behavior 50: 595–603. 36511085 10.1177/10901981221139179 PMC10468152

[bibr6-20551029251327438] Bachman AS Vanderpool RC Cohen E , et al. (2018) Stigma and uncertainty in media presentation of human papillomavirus. Kentucky Journal of Communication 37: 4–29.

[bibr7-20551029251327438] Bai J Belcher SM Meador R , et al. (2021) Comparisons of depression, sexual function, and quality of life between women with gynecological cancers and race-matched healthy controls. Cancer Nursing 44: 116–124. 31569179 10.1097/NCC.0000000000000744

[bibr8-20551029251327438] Bartoces MG Severson RK Rusin BA , et al. (2009) Quality of life and self-esteem of long-term survivors of invasive and noninvasive cervical cancer. Journal of Women’s Health 18: 655–661. 10.1089/jwh.2008.0959 PMC2851133 19405862

[bibr9-20551029251327438] Bauman JR El-Jawahri A Quinn K , et al. (2016) Patient reported outcomes (PROs) in patients with human papilloma virus (HPV) positive versus negative head and neck cancer (HNC). Journal of Clinical Oncology 34: 87.

[bibr10-20551029251327438] Baxi SS Shuman AG Corner GW , et al. (2013) Sharing a diagnosis of HPV‐related head and neck cancer: the emotions, the confusion, and what patients want to know. Head & Neck 35: 1534–1541. 23169350 10.1002/hed.23182 PMC3689851

[bibr11-20551029251327438] Baxi SS Salz T Xiao H , et al. (2016) Employment and return to work following chemoradiation in patient with HPV-related oropharyngeal cancer. Cancers of the Head & Neck 1: 4–8. 31093334 10.1186/s41199-016-0002-0 PMC6457145

[bibr12-20551029251327438] Baxi SS Cullen G Xiao H , et al. (2018) Long‐term quality of life in older patients with HPV‐related oropharyngeal cancer. Head & Neck 40: 2321–2328. 30421835 10.1002/hed.25159 PMC6681446

[bibr13-20551029251327438] Baze C Monk BJ Herzog TJ (2008) The impact of cervical cancer on quality of life: a personal account. Gynecologic Oncology 109: S12–S14. 18482554 10.1016/j.ygyno.2008.01.022

[bibr14-20551029251327438] Beeler WH Bellile EL Casper KA , et al. (2020) Patient-reported financial toxicity and adverse medical consequences in head and neck cancer. Oral Oncology 101: 104521. 31877502 10.1016/j.oraloncology.2019.104521 PMC7008081

[bibr15-20551029251327438] Best AL Logan RG Vázquez-Otero C , et al. (2018) Application of a health literacy framework to explore patients’ knowledge of the link between HPV and cancer. Journal of Health Communication 23: 1–8. 30153087 10.1080/10810730.2018.1508257 PMC6413510

[bibr16-20551029251327438] Boehmer U Miao X Ozonoff A (2011) Cancer survivorship and sexual orientation. Cancer 117: 3796–3804. 21557209 10.1002/cncr.25950

[bibr17-20551029251327438] Brown B Poteat T Marg L , et al. (2017) Human papillomavirus-related cancer surveillance, prevention, and screening among transgender men and women: neglected populations at high risk. LGBT Health 4: 315–319. 28876211 10.1089/lgbt.2016.0142

[bibr18-20551029251327438] Brown Johnson CG Brodsky JL Cataldo JK (2014) Lung cancer stigma, anxiety, depression, and quality of life. Journal of Psychosocial Oncology 32: 59–73. 24428251 10.1080/07347332.2013.855963 PMC4634635

[bibr19-20551029251327438] Bruni L Albero G Rowley J , et al. (2023) Global and regional estimates of genital human papillomavirus prevalence among men: a systematic review and meta-analysis. Lancet Global Health 11: e1345–e1362. 37591583 10.1016/S2214-109X(23)00305-4 PMC10447222

[bibr20-20551029251327438] Cardoso RC Kamal M Zaveri J , et al. (2021) Self-reported trismus: prevalence, severity and impact on quality of life in oropharyngeal cancer survivorship: a cross-sectional survey report from a comprehensive cancer center. Supportive Care in Cancer 29: 1825–1835. 32779007 10.1007/s00520-020-05630-7 PMC8790744

[bibr21-20551029251327438] Carter J Sonoda Y Chi DS , et al. (2008) Radical trachelectomy for cervical cancer: postoperative physical and emotional adjustment concerns. Gynecologic Oncology 111: 151–157. 18662827 10.1016/j.ygyno.2008.06.003

[bibr22-20551029251327438] Carter J Sonoda Y Baser RE , et al. (2010) A 2-year prospective study assessing the emotional, sexual, and quality of life concerns of women undergoing radical trachelectomy versus radical hysterectomy for treatment of early-stage cervical cancer. Gynecologic Oncology 119: 358–365. 20817227 10.1016/j.ygyno.2010.07.016 PMC4847134

[bibr23-20551029251327438] CENTERS FOR DISEASE CONTROL AND PREVENTION (2021a) Cancers Associated with Human Papillomavirus, United States—2014–2018 [Online]. USCS Data Brief. https://www.cdc.gov/cancer/uscs/about/data-briefs/no26-hpv-assoc-cancers-UnitedStates-2014-2018.htm [Accessed 4/6/2022].

[bibr24-20551029251327438] CENTERS FOR DISEASE CONTROL AND PREVENTION (2021b) How Many Cancers Are Linked with HPV Each Year? [Online]. Available. https://www.cdc.gov/cancer/hpv/statistics/cases.htm#3

[bibr25-20551029251327438] Chakoma T Moon PK Osazuwa-Peters OL , et al. (2023) Association of human papillomavirus status with suicide risk among patients with head and neck cancer. JAMA Otolaryngology–Head & Neck Surgery 149: 291–299. 36795392 10.1001/jamaoto.2022.4839 PMC9936382

[bibr26-20551029251327438] Chambers SK Baade P Youl P , et al. (2015) Psychological distress and quality of life in lung cancer: the role of health‐related stigma, illness appraisals and social constraints. Psycho-Oncology 24: 1569–1577. 25920906 10.1002/pon.3829 PMC5029590

[bibr27-20551029251327438] Chan JL Letourneau J Salem W , et al. (2015) Sexual satisfaction and quality of life in survivors of localized cervical and ovarian cancers following fertility-sparing surgery. Gynecologic Oncology 139: 141–147. 26232519 10.1016/j.ygyno.2015.07.105

[bibr28-20551029251327438] Chaturvedi AK Graubard BI Broutian T , et al. (2018) Effect of prophylactic human papillomavirus (HPV) vaccination on oral HPV infections among young adults in the United States. Journal of Clinical Oncology 36: 262–267. 29182497 10.1200/JCO.2017.75.0141 PMC5773841

[bibr29-20551029251327438] Chido-Amajuoyi OG Jackson I Yu R , et al. (2020) Declining Awareness of HPV and HPV Vaccination within the General US Population. AACR. 10.1080/21645515.2020.1783952 PMC7899652 32692632

[bibr30-20551029251327438] Cho J Choi EK Kim SY , et al. (2013) Association between cancer stigma and depression among cancer survivors: a nationwide survey in Korea. Psycho-Oncology 22: 2372–2378. 23784964 10.1002/pon.3302

[bibr31-20551029251327438] Clemmens DA Knafl K Lev EL , et al. (2008) Cervical cancer: patterns of long-term survival. Oncology Nursing Forum 35: 897–903. 18980920 10.1188/08.ONF.897-903

[bibr32-20551029251327438] Coleman D Hurtado-de-Mendoza A Montero A , et al. (2022) Stigma, social support, and spirituality: associations with symptoms among Black, Latina, and Chinese American cervical cancer survivors. Journal of Cancer Survivorship 18: 710–726. 36417116 10.1007/s11764-022-01283-z PMC10200827

[bibr33-20551029251327438] D’Souza G Wentz A Kluz N , et al. (2016) Sex differences in risk factors and natural history of oral human papillomavirus infection. The Journal of Infectious Diseases 213: 1893–1896. 26908748 10.1093/infdis/jiw063 PMC9136851

[bibr34-20551029251327438] Daley EM Vamos CA Wheldon CW , et al. (2015) Negative emotions and stigma associated with a human papillomavirus test result: a comparison between human papillomavirus–positive men and women. Journal of Health Psychology 20: 1073–1082. 24217064 10.1177/1359105313507963

[bibr35-20551029251327438] Daley EM Vamos CA Thompson EL , et al. (2017) The feminization of HPV: how science, politics, economics and gender norms shaped US HPV vaccine implementation. Papillomavirus Research 3: 142–148. 28720448 10.1016/j.pvr.2017.04.004 PMC5883212

[bibr36-20551029251327438] Dodd RH Forster AS Marlow LA , et al. (2019) Psychosocial impact of human papillomavirus‐related head and neck cancer on patients and their partners: a qualitative interview study. European Journal of Cancer Care 28: e12999. 30677190 10.1111/ecc.12999 PMC6559265

[bibr37-20551029251327438] Doll KM Barber EL Bensen JT , et al. (2016) The impact of surgical complications on health-related quality of life in women undergoing gynecologic and gynecologic oncology procedures: a prospective longitudinal cohort study. American Journal of Obstetrics and Gynecology 215: 457. 10.1016/j.ajog.2016.04.025 PMC5573237 27131589

[bibr38-20551029251327438] Donovan KA Taliaferro LA Alvarez EM , et al. (2007) Sexual health in women treated for cervical cancer: characteristics and correlates. Gynecologic Oncology 104: 428–434. 17005248 10.1016/j.ygyno.2006.08.009

[bibr39-20551029251327438] Drake VE Fakhry C Windon MJ , et al. (2021) Timing, number, and type of sexual partners associated with risk of oropharyngeal cancer. Cancer 127: 1029–1038. 33426652 10.1002/cncr.33346 PMC8035131

[bibr40-20551029251327438] Duhamel K Schuler T Nelson C , et al. (2016) The sexual health of female rectal and anal cancer survivors: results of a pilot randomized psycho-educational intervention trial. Journal of Cancer Survivorship 10: 553–563. 26667358 10.1007/s11764-015-0501-8 PMC4864056

[bibr41-20551029251327438] Dyer KE (2010) From cancer to sexually transmitted infection: explorations of social stigma among cervical cancer survivors. Human Organization 69: 321–330.

[bibr42-20551029251327438] Ell K Xie B Wells A , et al. (2008) Economic stress among low‐income women with cancer: effects on quality of life. Cancer: Interdisciplinary International Journal of the American Cancer Society 112: 616–625. 10.1002/cncr.23203 18085642

[bibr43-20551029251327438] Ernst J Mehnert A Dietz A , et al. (2017) Perceived stigmatization and its impact on quality of life-results from a large register-based study including breast, colon, prostate and lung cancer patients. BMC Cancer 17: 1–8. 29121876 10.1186/s12885-017-3742-2 PMC5680772

[bibr44-20551029251327438] Finneran C Johnson Peretz J Blemur D , et al. (2021) “That’s only for women”: the importance of educating HIV-positive sexual minority men on HPV and high resolution anoscopy (HRA). Journal of the International Association of Providers of AIDS Care (JIAPAC) 20: 23259582211016134. 34056930 10.1177/23259582211016134 PMC8170352

[bibr45-20551029251327438] Fleming N Ramirez P Soliman P , et al. (2016) Quality of life after radical trachelectomy for early-stage cervical cancer: a 5-year prospective evaluation. Gynecologic Oncology 143: 596–603. 27742473 10.1016/j.ygyno.2016.10.012 PMC5439265

[bibr46-20551029251327438] Fujisawa D Hagiwara N (2015) Cancer stigma and its health consequences. Current Breast Cancer Reports 7: 143–150.

[bibr47-20551029251327438] Goepfert RP Fuller CD Gunn GB , et al. (2017) Symptom burden as a driver of decisional regret in long‐term oropharyngeal carcinoma survivors. Head & Neck 39: 2151–2158. 28736965 10.1002/hed.24879

[bibr48-20551029251327438] Gonzales G Zinone R (2018) Cancer diagnoses among lesbian, gay, and bisexual adults: results from the 2013–2016 National Health Interview Survey. Cancer Causes & Control 29: 845–854. 30043193 10.1007/s10552-018-1060-x

[bibr49-20551029251327438] Gotay CC Farley JH Kawamoto CT , et al. (2008) Adaptation and quality of life among long-term cervical cancer survivors in the military health care system. Military Medicine 173: 1035–1041. 19160626 10.7205/milmed.173.10.1035

[bibr50-20551029251327438] Greenwald HP Mccorkle R (2007) Remedies and life changes among invasive cervical cancer survivors. Urologic Nursing 27: 47–53. 17390927

[bibr51-20551029251327438] Greenwald HP Mccorkle R (2008) Sexuality and sexual function in long-term survivors of cervical cancer. Journal of Women’s Health 17: 955–963. 10.1089/jwh.2007.0613 PMC2942787 18681816

[bibr52-20551029251327438] Greenwald HP Mccorkle R Fennie K (2008) Health status and adaptation among long-term cervical cancer survivors. Gynecologic Oncology 111: 449–454. 18835023 10.1016/j.ygyno.2008.08.015

[bibr53-20551029251327438] Guerendiain D Grigorescu R Kirk A , et al. (2022) HPV status and HPV16 viral load in anal cancer and its association with clinical outcome. Cancer Medicine 11: 4193–4203. 35785486 10.1002/cam4.4771 PMC9678095

[bibr54-20551029251327438] Hammermüller C Hinz A Dietz A , et al. (2021) Depression, anxiety, fatigue, and quality of life in a large sample of patients suffering from head and neck cancer in comparison with the general population. BMC Cancer 21: 1–11. 33482771 10.1186/s12885-020-07773-6 PMC7825198

[bibr55-20551029251327438] Heck JE Berthiller J Vaccarella S , et al. (2010) Sexual behaviours and the risk of head and neck cancers: a pooled analysis in the International Head and Neck Cancer Epidemiology (INHANCE) consortium. International Journal of Epidemiology 39: 166–181. 20022926 10.1093/ije/dyp350 PMC2817092

[bibr56-20551029251327438] Hill EK Sandbo S Abramsohn E , et al. (2011) Assessing gynecologic and breast cancer survivors’ sexual health care needs. Cancer 117: 2643–2651. 21656742 10.1002/cncr.25832 PMC3084902

[bibr57-20551029251327438] Hirth J (2019) Disparities in HPV vaccination rates and HPV prevalence in the United States: a review of the literature. Human Vaccines & Immunotherapeutics 15: 146–155. 30148974 10.1080/21645515.2018.1512453 PMC6363146

[bibr58-20551029251327438] Hoover DS Spears CA Vidrine DJ , et al. (2019) Smoking cessation treatment needs of low SES cervical cancer survivors. American Journal of Health Behavior 43: 606–620. 31046890 10.5993/AJHB.43.3.14 PMC6686858

[bibr59-20551029251327438] Huang Z Yu T Wu S , et al. (2021) Correlates of stigma for patients with cancer: a systematic review and meta-analysis. Supportive Care in Cancer 29: 1195–1203. 32951087 10.1007/s00520-020-05780-8

[bibr60-20551029251327438] Hubbs J Dickson Michelson EL Vogel R , et al. (2019) Sexual quality of life after the treatment of gynecologic cancer: what women want. Supportive Care in Cancer 27: 4649–4654. 30941579 10.1007/s00520-019-04756-7 PMC6774914

[bibr61-20551029251327438] Iyer NS Osann K Hsieh S , et al. (2016) Health behaviors in cervical cancer survivors and associations with quality of life. Clinical Therapeutics 38: 467–475. 26926320 10.1016/j.clinthera.2016.02.006 PMC4799758

[bibr62-20551029251327438] Janz TA Momin SR Sterba KR , et al. (2019) Comparison of psychosocial factors over time among HPV+ oropharyngeal cancer and tobacco-related oral cavity cancer patients. American Journal of Otolaryngology 40: 40–45. 30322742 10.1016/j.amjoto.2018.08.010

[bibr63-20551029251327438] Kaffenberger TM Patel AK Lyu L , et al. (2021) Quality of life after radiation and transoral robotic surgery in advanced oropharyngeal cancer. Laryngoscope Investigative Otolaryngology 6: 983–990. 34667840 10.1002/lio2.628 PMC8513430

[bibr64-20551029251327438] Kepka D Ding Q Hawkins AJ , et al. (2016) Factors associated with early adoption of the HPV vaccine in US male adolescents include Hispanic ethnicity and receipt of other vaccines. Preventive Medicine Reports 4: 98–102. 27413668 10.1016/j.pmedr.2016.05.014 PMC4929120

[bibr65-20551029251327438] Kim YJ Munsell MF Park JC , et al. (2015) Retrospective review of symptoms and palliative care interventions in women with advanced cervical cancer. Gynecologic Oncology 139: 553–558. 26432043 10.1016/j.ygyno.2015.09.079 PMC8765286

[bibr66-20551029251327438] Koenig LJ Hoyer D Purcell DW , et al. (2016) Young people and HIV: a call to action. American Journal of Public Health 106: 402–405. 26794156 10.2105/AJPH.2015.302979 PMC4815747

[bibr67-20551029251327438] Kompelli A Cartmell KB Sterba KR , et al. (2020) An assessment of racial differences in epidemiological, clinical and psychosocial factors among head and neck cancer patients at the time of surgery. World Journal of Otorhinolaryngology-Head and Neck Surgery 6: 41–48. 32426702 10.1016/j.wjorl.2019.01.002 PMC7221208

[bibr68-20551029251327438] Kugelman N Siegler E Mackuli L , et al. (2022) Prognosis of human papillomavirus–negative compared to human papillomavirus–positive cervical cancer. Journal of Lower Genital Tract Disease 26: 115–121. 34967775 10.1097/LGT.0000000000000650

[bibr69-20551029251327438] Lathan CS Cronin A Tucker-Seeley R , et al. (2016) Association of financial strain with symptom burden and quality of life for patients with lung or colorectal cancer. Journal of Clinical Oncology 34: 1732–1740. 26926678 10.1200/JCO.2015.63.2232 PMC4966336

[bibr70-20551029251327438] Lebel S Devins GM (2008) Stigma in cancer patients whose behavior may have contributed to their disease. 10.2217/14796694.4.5.717 18922128

[bibr71-20551029251327438] Lebel S Castonguay M Mackness G , et al. (2013) The psychosocial impact of stigma in people with head and neck or lung cancer. Psycho-Oncology 22: 140–152. 21932417 10.1002/pon.2063

[bibr72-20551029251327438] Lechner M Liu J Masterson L , et al. (2022) HPV-associated oropharyngeal cancer: epidemiology, molecular biology and clinical management. Nature Reviews Clinical Oncology 19: 306–327. 10.1038/s41571-022-00603-7 PMC8805140 35105976

[bibr73-20551029251327438] Leppard S (2016) Anal cancer is on the rise; it’s a shame. Australian Family Physician 45: 252–253. 27052148

[bibr74-20551029251327438] Levac D Colquhoun H O’Brien KK (2010) Scoping studies: advancing the methodology. Implementation Science 5: 1–9. 20854677 10.1186/1748-5908-5-69 PMC2954944

[bibr75-20551029251327438] Liao C-I Francoeur AA Kapp DS , et al. (2022) Trends in human papillomavirus–associated cancers, demographic characteristics, and vaccinations in the US, 2001-2017. JAMA Network Open 5: e222530. 35294540 10.1001/jamanetworkopen.2022.2530 PMC8928005

[bibr76-20551029251327438] Lin C Slama J Gonzalez P , et al. (2019) Cervical determinants of anal HPV infection and high-grade anal lesions in women: a collaborative pooled analysis. The Lancet Infectious Diseases 19: 880–891. 31204304 10.1016/S1473-3099(19)30164-1 PMC6656696

[bibr77-20551029251327438] Lindau ST Gavrilova N Anderson D (2007) Sexual morbidity in very long term survivors of vaginal and cervical cancer: a comparison to national norms. Gynecologic Oncology 106: 413–418. 17582473 10.1016/j.ygyno.2007.05.017 PMC2716652

[bibr78-20551029251327438] Ling D Chapman B Kim J , et al. (2016) Oncologic outcomes and patient-reported quality of life in patients with oropharyngeal squamous cell carcinoma treated with definitive transoral robotic surgery versus definitive chemoradiation. Oral Oncology 61: 41–46. 27688103 10.1016/j.oraloncology.2016.08.004 PMC7717075

[bibr79-20551029251327438] Linsky A Nyambose J Battaglia TA (2011) Lifestyle behaviors in Massachusetts adult cancer survivors. Journal of Cancer Survivorship 5: 27–34. 21132395 10.1007/s11764-010-0162-6

[bibr80-20551029251327438] Longabaugh M (2017) Patient perspective and personal journey of treating a “Rare Cancer”. Surgical Oncology Clinics 26: 1–7. 27889029 10.1016/j.soc.2016.07.014

[bibr81-20551029251327438] Mady LJ Lyu L Owoc MS , et al. (2019) Understanding financial toxicity in head and neck cancer survivors. Oral Oncology 95: 187–193. 31345389 10.1016/j.oraloncology.2019.06.023

[bibr82-20551029251327438] Markowitz LE Gee J Chesson H , et al. (2018) Ten years of human papillomavirus vaccination in the United States. Academic Pediatrics 18: S3–S10. 29502635 10.1016/j.acap.2017.09.014 PMC11331487

[bibr83-20551029251327438] Massa ST Osazuwa-Peters N Adjei Boakye E , et al. (2019) Comparison of the financial burden of survivors of head and neck cancer with other cancer survivors. JAMA Otolaryngology–Head & Neck Surgery 145: 239–249. 30789634 10.1001/jamaoto.2018.3982 PMC6439752

[bibr84-20551029251327438] Maxwell JH Mehta V Wang H , et al. (2014) Quality of life in head and neck cancer patients: impact of HPV and primary treatment modality. The Laryngoscope 124: 1592–1597. 24353066 10.1002/lary.24508

[bibr85-20551029251327438] Mccaffery K Waller J Nazroo J , et al. (2006) Social and psychological impact of HPV testing in cervical screening: a qualitative study. Sexually Transmitted Infections 82: 169–174. 16581749 10.1136/sti.2005.016436 PMC2564695

[bibr86-20551029251327438] Milbury K Rosenthal DI El-Naggar A , et al. (2013) An exploratory study of the informational and psychosocial needs of patients with human papillomavirus-associated oropharyngeal cancer. Oral Oncology 49: 1067–1071. 23953777 10.1016/j.oraloncology.2013.07.010 PMC3964874

[bibr87-20551029251327438] Moreno KF Khabbaz E Gaitonde K , et al. (2012) Sexuality after treatment of head and neck cancer: findings based on modification of sexual adjustment questionnaire. The Laryngoscope 122: 1526–1531. 22508246 10.1002/lary.23347

[bibr88-20551029251327438] Moscicki A-B Palefsky JM (2011) Human papillomavirus in men: an update. Journal of Lower Genital Tract Disease 15: 231–234. 21543996 10.1097/LGT.0b013e318203ae61 PMC3304470

[bibr89-20551029251327438] Neugut AI Subar M Wilde ET , et al. (2011) Association between prescription co-payment amount and compliance with adjuvant hormonal therapy in women with early-stage breast cancer. Journal of Clinical Oncology 29: 2534–2542. 21606426 10.1200/JCO.2010.33.3179 PMC3138633

[bibr90-20551029251327438] Osann K Hsieh S Nelson EL , et al. (2014) Factors associated with poor quality of life among cervical cancer survivors: implications for clinical care and clinical trials. Gynecologic Oncology 135: 266–272. 25192629 10.1016/j.ygyno.2014.08.036 PMC4479396

[bibr91-20551029251327438] Osazuwa-Peters N Adjei Boakye E Mohammed KA , et al. (2017) Not just a woman’s business! Understanding men and women’s knowledge of HPV, the HPV vaccine, and HPV-associated cancers. Preventive Medicine 99: 299–304. 28341458 10.1016/j.ypmed.2017.03.014

[bibr92-20551029251327438] Ostroff JS Riley KE Shen MJ , et al. (2019) Lung cancer stigma and depression: validation of the lung cancer stigma inventory. Psycho-Oncology 28: 1011–1017. 30779396 10.1002/pon.5033 PMC6506363

[bibr93-20551029251327438] Penson RT Wenzel LB Vergote I , et al. (2006) Quality of life considerations in gynecologic cancer. International Journal of Gynecology & Obstetrics 95: S247–S257. 10.1016/S0020-7292(06)60040-4 29644666

[bibr94-20551029251327438] Peters MD Godfrey CM Khalil H , et al. (2015) Guidance for conducting systematic scoping reviews. JBI Evidence Implementation 13: 141–146. 10.1097/XEB.0000000000000050 26134548

[bibr95-20551029251327438] Phelan SM Griffin JM Jackson GL , et al. (2013) Stigma, perceived blame, self‐blame, and depressive symptoms in men with colorectal cancer. Psycho-Oncology 22: 65–73. 21954081 10.1002/pon.2048 PMC6000725

[bibr96-20551029251327438] Qualliotine J Califano J Li R , et al. (2017) Human papillomavirus tumour status is not associated with a positive depression screen for patients with oropharyngeal cancer. Journal of Laryngology & Otology 131: 760–767. 28720154 10.1017/S0022215117001098

[bibr97-20551029251327438] Quinn GP Sanchez JA Sutton SK , et al. (2015) Cancer and lesbian, gay, bisexual, transgender/transsexual, and queer/questioning (LGBTQ) populations. CA: A Cancer Journal for Clinicians 65: 384–400. 26186412 10.3322/caac.21288 PMC4609168

[bibr98-20551029251327438] Ramsey S Blough D Kirchhoff A , et al. (2013) Washington State cancer patients found to be at greater risk for bankruptcy than people without a cancer diagnosis. Health Affairs 32: 1143–1152. 23676531 10.1377/hlthaff.2012.1263 PMC4240626

[bibr99-20551029251327438] Ramsey SD Bansal A Fedorenko CR , et al. (2016) Financial insolvency as a risk factor for early mortality among patients with cancer. Journal of Clinical Oncology 34: 980–986. 26811521 10.1200/JCO.2015.64.6620 PMC4933128

[bibr100-20551029251327438] Rhoten BA Davis AJ Baraff BN , et al. (2020) Priorities and preferences of patients with head and neck cancer for discussing and receiving information about sexuality and perception of self-report measures. The Journal of Sexual Medicine 17: 1529–1537. 32417203 10.1016/j.jsxm.2020.04.001 PMC7664992

[bibr101-20551029251327438] Roick J Danker H Kersting A , et al. (2019) The association of socioeconomic status with quality of life in cancer patients over a 6-month period using individual growth models. Supportive Care in Cancer 27: 3347–3355. 30627920 10.1007/s00520-018-4634-y

[bibr102-20551029251327438] Saleem AM (2011) Risk of Anal Cancer in a Cohort with Human Papillomavirus-Related Gynecologic Neoplasm. Sackler School of Graduate Biomedical Sciences (Tufts University). 10.1097/AOG.0b013e31820bfb16 21343768

[bibr103-20551029251327438] Shepherd MA Gerend MA (2014) The blame game: cervical cancer, knowledge of its link to human papillomavirus and stigma. Psychology and Health 29: 94–109. 10.1080/08870446.2013.834057 24006882

[bibr104-20551029251327438] Silver JA Schwartz R Roy CF , et al. (2023) Patient-reported outcome measures of psychosocial quality of life in oropharyngeal cancer patients: a scoping review. Journal of Clinical Medicine 12: 2122. 36983125 10.3390/jcm12062122 PMC10057395

[bibr105-20551029251327438] Simon MA de la Riva EE Bergan R , et al. (2014) Improving diversity in cancer research trials: the story of the Cancer Disparities Research Network. Journal of Cancer Education 29: 366–374. 24519744 10.1007/s13187-014-0617-y PMC4029870

[bibr106-20551029251327438] Slade BA Leidel L Vellozzi C , et al. (2009) Postlicensure safety surveillance for quadrivalent human papillomavirus recombinant vaccine. JAMA 302: 750–757. 19690307 10.1001/jama.2009.1201

[bibr107-20551029251327438] Stanley MA Winder DM Sterling JC , et al. (2012) HPV infection, anal intra-epithelial neoplasia (AIN) and anal cancer: current issues. BMC Cancer 12: 1–4. 22958276 10.1186/1471-2407-12-398 PMC3488563

[bibr108-20551029251327438] Taberna M Inglehart RC Pickard RK , et al. (2017) Significant changes in sexual behavior after a diagnosis of human papillomavirus‐positive and human papillomavirus‐negative oral cancer. Cancer 123: 1156–1165. 28195638 10.1002/cncr.30564

[bibr109-20551029251327438] Van Dyne EA Henley SJ Saraiya M , et al. (2018) Trends in human papillomavirus–associated cancers—United States, 1999–2015. Morbidity and Mortality Weekly Report 67: 918–924. 30138307 10.15585/mmwr.mm6733a2 PMC6107321

[bibr110-20551029251327438] Walsh T Bertozzi-Villa C Schneider JA (2015) Systematic review of racial disparities in human papillomavirus–associated anal dysplasia and anal cancer among men who have sex with men. American Journal of Public Health 105: e34–e45. 25713941 10.2105/AJPH.2014.302469 PMC4358202

[bibr111-20551029251327438] Wang MB Liu IY Gornbein JA , et al. (2015) HPV-positive oropharyngeal carcinoma: a systematic review of treatment and prognosis. Otolaryngology-Head and Neck Surgery 153: 758–769. 26124261 10.1177/0194599815592157

[bibr112-20551029251327438] Wearn A Shepherd L (2020) The impact of emotion‐based mass media campaigns on stigma toward cervical screening non participation. Journal of Applied Social Psychology 50: 289–298.

[bibr113-20551029251327438] Wenzel L Osann K Hsieh S , et al. (2015) Psychosocial telephone counseling for survivors of cervical cancer: results of a randomized biobehavioral trial. Journal of Clinical Oncology 33: 1171–1179. 25713429 10.1200/JCO.2014.57.4079 PMC4372853

[bibr114-20551029251327438] Westin SN Sun CC Tung CS , et al. (2016) Survivors of gynecologic malignancies: impact of treatment on health and well-being. Journal of Cancer Survivorship 10: 261–270. 26245979 10.1007/s11764-015-0472-9 PMC4744585

[bibr115-20551029251327438] Wilford J Osann K Hsieh S , et al. (2018) Validation of PROMIS emotional distress short form scales for cervical cancer. Gynecologic Oncology 151: 111–116. 30078504 10.1016/j.ygyno.2018.07.022 PMC6171766

[bibr116-20551029251327438] Wilson IB Cleary PD (1995) Linking clinical variables with health-related quality of life: a conceptual model of patient outcomes. JAMA 273: 59–65. 7996652

[bibr117-20551029251327438] Wilson CM Mcguire DB Rodgers BL , et al. (2020) Body image, sexuality, and sexual functioning in cervical and endometrial cancer: interrelationships and women’s experiences. Sexuality and Disability 38: 389–403.

[bibr118-20551029251327438] Windon MJ D’Souza G Faraji F , et al. (2019) Priorities, concerns, and regret among patients with head and neck cancer. Cancer 125: 1281–1289. 30645761 10.1002/cncr.31920 PMC6443481

[bibr119-20551029251327438] Wo JY Drapek LC Niemierko A , et al. (2018) Clinical needs assessment for sexual health among cancer patients receiving pelvic radiation: implications for development of a radiation oncology sexual health clinic. Practical Radiation Oncology 8: 206–212. 29426693 10.1016/j.prro.2017.11.004

[bibr120-20551029251327438] Xiao C Zhang Q Nguyen-Tân PF , et al. (2017) Quality of life and performance status from a substudy conducted within a prospective phase 3 randomized trial of concurrent standard radiation versus accelerated radiation plus cisplatin for locally advanced head and neck carcinoma: NRG Oncology RTOG 0129. International Journal of Radiation Oncology, Biology, Physics 97: 667–677. 27727063 10.1016/j.ijrobp.2016.07.020 PMC5266672

[bibr121-20551029251327438] Xu MJ Plonowska KA Gurman ZR , et al. (2020) Treatment modality impact on quality of life for human papillomavirus–associated oropharynx cancer. The Laryngoscope 130: E48–E56. 30919470 10.1002/lary.27937

[bibr122-20551029251327438] Ye Y Burkholder GA Wiener HW , et al. (2020) Comorbidities associated with HPV infection among people living with HIV-1 in the southeastern US: a retrospective clinical cohort study. BMC Infectious Diseases 20: 1–9. 10.1186/s12879-020-4822-5 PMC7023731 32059635

[bibr123-20551029251327438] Yilmaz M Dissiz G Usluoğlu AK , et al. (2020) Cancer-related stigma and depression in cancer patients in a middle-income country. Asia-Pacific Journal of Oncology Nursing 7: 95–102. 31879690 10.4103/apjon.apjon_45_19 PMC6927157

[bibr124-20551029251327438] Zafar SY Mcneil RB Thomas CM , et al. (2015) Population-based assessment of cancer survivors’ financial burden and quality of life: a prospective cohort study. Journal of Oncology Practice 11: 145–150. 25515717 10.1200/JOP.2014.001542 PMC4371118

