# Supplementary material for: Psychosocial aspects of quality of life outcomes in post-treatment human papillomavirus-associated cancer survivors in the United States: A scoping review
Source: Health Psychol Open. 2025 Mar 27;12:20551029251327438. doi: 10.1177/20551029251327438 (PMC11951441; doi:10.1177/20551029251327438)
Supplement: Supplemental Material - Psychosocial aspects of quality of life outcomes in post-treatment human papillomavirus-associated cancer survivors in the United States: A scoping review [file sj-pdf-1-hpo-10.1177_20551029251327438.pdf]

Supplement 1: List of search terms.

- "HPV" and "anal cancer"
- "HPV" and "rectal cancer"
- "HPV" and "anal cancer" and "rectal cancer"
- "HPV" and "penile cancer"
- "HPV" and "quality of life" and "anal cancer"
- "HPV" and "quality of life" and "rectal cancer"
- "HPV" and "quality of life" and "penile cancer"
- "HPV" and "psychosocial" and "anal cancer"
- "HPV" and "psychosocial" and "rectal cancer"
- "HPV" and "psychosocial" and "penile cancer"
- "HPV" and "anorectal cancer"
- "HPV" and "stigma" and "anal cancer"
- "HPV" and "stigma" and "rectal cancer"
- "HPV" and "stigma" and "anorectal cancer"
- "HPV" and "stigma" and "penile cancer"
- "HPV" and "depression" and "anal cancer"
- "HPV" and "depression" and "rectal cancer"
- "HPV" and "depression" and "anorectal cancer"
- "HPV" and "depression" and "penile cancer"
- "HPV" and "anxiety" and "anal cancer"
- "HPV" and "anxiety" and "rectal cancer"
- "HPV" and "anxiety" and "anorectal cancer"
- "HPV" and "anxiety" and "penile cancer"
- "HPV" and "mental health" and "anal cancer"
- "HPV" and "mental health" and "rectal cancer"
- "HPV" and "mental health" and "anorectal cancer"
- "HPV" and "mental health" and "penile cancer"
- "HPV" and "socioeconomic" and "anal cancer"
- "HPV" and "socioeconomic" and "rectal cancer"
- "HPV" and "socioeconomic" and "anorectal cancer"
- "HPV" and "socioeconomic" and "penile cancer"
- "HPV" and "sexual intimacy" and "anal cancer"
- "HPV" and "sexual intimacy" and "rectal cancer"
- "HPV" and "sexual intimacy" and "anorectal cancer"
- "HPV" and "sexual intimacy" and "penile cancer"
- "HPV" and "sexual function" and "anal cancer"
- "HPV" and "sexual function" and "rectal cancer"
- "HPV" and "sexual function" and "anorectal cancer"
- "HPV" and "sexual function" and "penile cancer"
- "HPV" and "vaginal cancer"

- “HPV” and “vulvar cancer”
- “HPV” and "cervical cancer" and "quality of life"
- “HPV cancer” and “Socioeconomic factors”
- "cervical cancer" and "socioeconomic"
- "HPV cancer" and "sociocultural"
- "HPV cancer" and "socioeconomic status OR poverty OR low income"
- “HPV” and “cervical cancer” and “stigma”
- “HPV” and “vaginal cancer” and “stigma”
- “HPV” and “vulvar cancer” and “stigma”
- “HPV” and "cervical cancer" and "sexual problems"
- “HPV” and “cervical cancer” and “sexuality”
- “HPV” and “cervical cancer" and "sex"
- "HPV" and "cervical cancer" and “mental health”
- "HPV cancer" and "mental health" and “stigma”
- "HPV" and "cervical cancer" and “depression”
- "HPV" and "cervical cancer" and “anxiety”
- "HPV cancer” and “socioeconomic” and “sexuality”
- "HPV cancer" and "socioeconomic status OR poverty OR low income" and “stigma”
- "HPV cancer" and "vaginal cancer" and “sexuality”
- "HPV" and "vaginal cancer" and “anxiety”
- "HPV" and "vaginal cancer" and “socioeconomic”
- "cervical cancer" and "socioeconomic" and “depression”
- "cervical cancer" and "socioeconomic" and “anxiety”
- "cervical cancer" and "quality of life" and “anxiety”
- "cervical cancer" and "quality of life" and “depression”
- "cervical cancer" and "quality of life" and “sexual function”
- "cervical cancer" and "quality of life" and “sexuality”
- "cervical cancer" and "socioeconomic" and “stigma”
- "cervical cancer" and "sexuality" and “stigma”
- "HPV cancer" and "sexuality" and “stigma”
- "HPV cancer" and "socioeconomic" and “psychological effects”
- "HPV" and "cervical cancer” and “psychological effects”
- "HPV" and "cervical cancer” and “mental health”
- "HPV" and "cervical cancer” and “socioeconomic”
- "HPV" and "cervical cancer” and “sociocultural”
- "HPV" and "cervical cancer” and “psychosocial”
- “HPV cancer” and “quality of life”
- “HPV cancer” and “depression”
- “HPV cancer” and “psychosocial”
- “HPV cancer” and “psychology”
- “HPV cancer” and “anxiety”
- “HPV cancer” and “stress”

- “HPV cancer” and “Sexual behavior”
- “HPV cancer” and “Socioeconomic factors”
- “HPV cancer” and “Sociodemographic”
- “HPV cancer” and “stigma”
- “HPV cancer” and “fear”
- “HPV” and “oropharyngeal cancer” and “quality of life”
- “HPV” and “oropharyngeal cancer” and “depression”
- “HPV” and “oropharyngeal cancer” and “anxiety”
- “HPV” and “oropharyngeal cancer” and “psychosocial”
- “HPV” and “oropharyngeal cancer” and “psychology”
- “HPV” and “oropharyngeal cancer” and “stress”
- “HPV” and “oropharyngeal cancer” and “culture”
- “HPV” and “oropharyngeal cancer” and “Sexual behavior”
- “HPV” and “oropharyngeal cancer” and “sexual intimacy”
- “HPV” and “oropharyngeal cancer” and “sexual function”
- “HPV” and “oropharyngeal cancer” and “Socioeconomic factors”
- “HPV” and “oropharyngeal cancer” and “Sociodemographic”
- “HPV” and “oropharyngeal cancer” and “stigma”
- “HPV” and “oropharyngeal cancer” and “fear”
- “HPV” and “oropharyngeal cancer” and “religiosity”
- “HPV” and “oropharyngeal cancer” and “faith”
